# Neoadjuvant therapy for hepatocellular carcinoma: emerging combinations and biomarker challenges

**DOI:** 10.3389/fimmu.2026.1809834

**Published:** 2026-07-06

**Authors:** Suruiya Palihati, Hanjun Mo, Xiaocui Ye, Cheng Yi, Xu Liang

**Affiliations:** 1 Division of Abdominal Tumor Multimodality Treatment, Cancer Center, West China Hospital, Sichuan University, Sichuan, China; 2 Department of Radiation Oncology, Zhongshan Hospital, Fudan University, Shanghai, China; 3 Department of Radiology, Sichuan Clinical Research Center for Cancer, Sichuan Cancer Hospital & Institute, Sichuan Cancer Center, University of Electronic Science and Technology of China, Chengdu, China

**Keywords:** biomarkers, hepatocellular carcinoma, immunotherapy, interventional therapy, neoadjuvant therapy

## Abstract

**Background:**

Hepatocellular carcinoma (HCC) remains a leading cause of cancer-related mortality globally, with high recurrence rates limiting the success of curative treatments. Neoadjuvant therapy has recently arisen as a promising strategy to enhance surgical outcomes and survival. This review focuses specifically on neoadjuvant therapy for resectable HCC.

**Main findings:**

We summarize current neoadjuvant approaches in this defined setting, focusing on systemic therapies—including targeted agents, immune checkpoint inhibitors, and their combinations—as well as locoregional modalities such as transarterial chemoembolization (TACE), hepatic arterial infusion chemotherapy (HAIC), ablation, and radiotherapy. Systemic–locoregional combinations have demonstrated improved pathological response rates and event-free survival, with phase 2/3 trials such as CARES-009 providing the highest level of evidence to date. We further summarize progress in biomarkers for predicting treatment efficacy across molecular, cellular, histopathological, and imaging dimensions. Key advances include baseline immune activation signatures, dynamic serum biomarkers such as alpha-fetoprotein (AFP) kinetics and liquid biopsy indicators, and emerging radiomic signatures and spatial multi-omics approaches. We also highlight ongoing clinical trials that are evaluating novel combination regimens and biomarker-driven patient stratification.

**Conclusions:**

By identifying critical challenges in standardizing pathological response criteria, optimizing treatment duration and sequencing, and validating multimodal biomarkers, this review emphasizes the pivotal role of neoadjuvant therapy in reshaping curative-intent management for HCC and delineates actionable directions for future research.

## 1 Introduction

**Figure 1:**
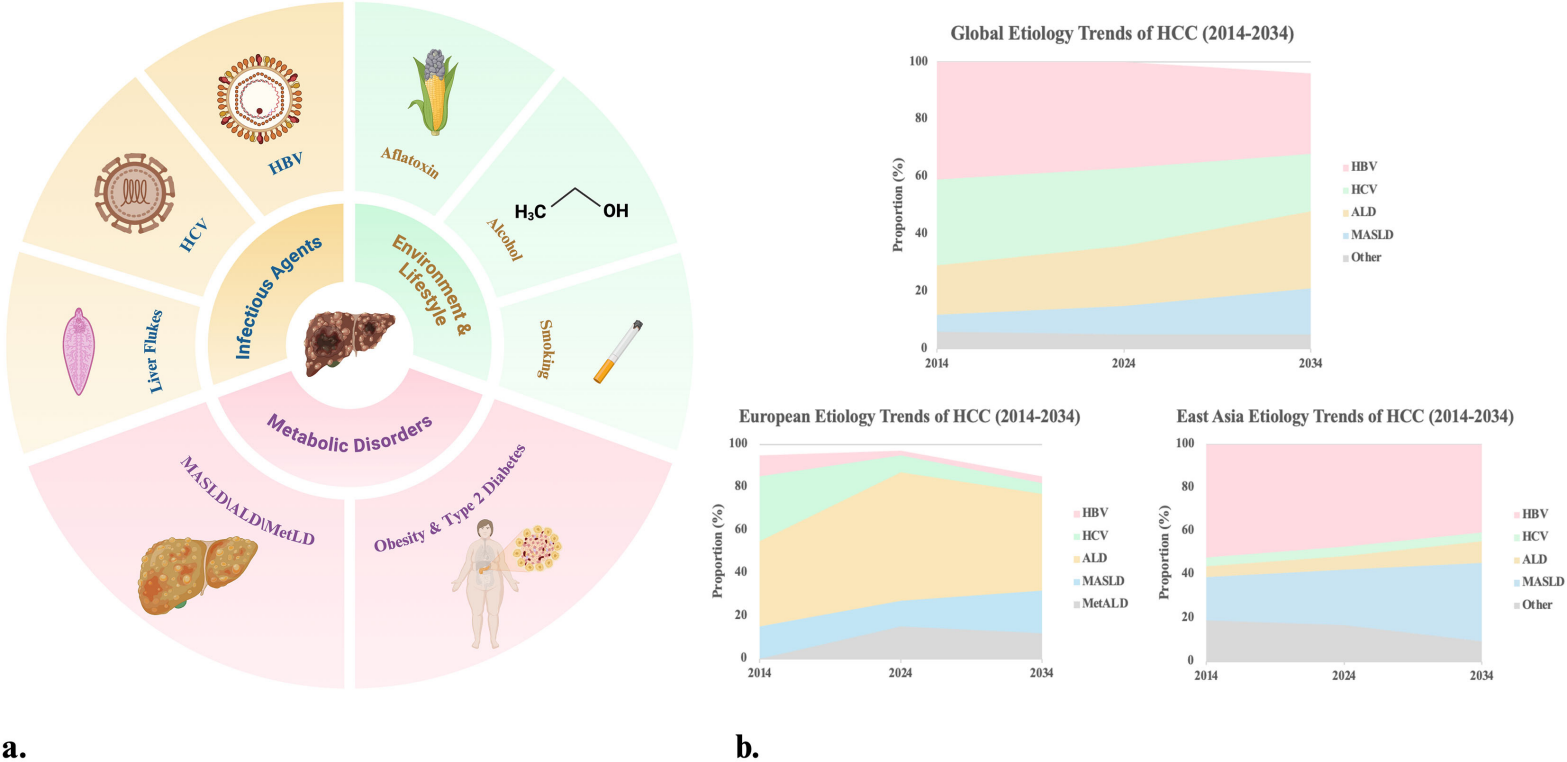
Major Risk Factors **(a)** and Etiological Shifts in HCC with Projected Trends (2014–2034) **(b)**. **(a)** HCC arises from diverse etiological factors broadly categorized into infectious agents, environmental/lifestyle exposures, and metabolic disorders. Chronic viral hepatitis, particularly hepatitis B virus (HBV) and hepatitis C virus (HCV), remains a dominant global driver. Liver fluke infections (Opisthorchis viverrini, Clonorchis sinensis), endemic in Southeast Asia, are significant carcinogens. Environmental exposures include potent dietary toxins like aflatoxin B1, which synergizes strongly with HBV, and lifestyle factors such as chronic alcohol consumption (promoting cirrhosis) and smoking. Concurrently, metabolic disorders – including obesity, type 2 diabetes, and metabolic dysfunction-associated steatotic liver disease (MASLD) – are rapidly emerging as critical and prevalent contributors to the HCC burden worldwide. **(b)** Figure presents the shifts and the projected trends in the etiology of HCC globally, in Europe, and in East Asia from 2014 to 2034. The area plots illustrate the proportion of HCC cases attributed to different etiologies, including HBV, HCV, alcohol-associated liver disease (ALD), MASLD, Metabolic dysfunction and alcohol-associated liver disease (MetALD) with increased alcohol intake, and other causes. The global trend shows a gradual increase in the proportion of HCC cases related to MASLD and MetALD, while the proportion attributed to HBV and HCV is projected to decrease. In Europe, similar trends are observed, with a notable rise in MASLD and MetALD-related HCC. Conversely, in East Asia, HBV remains the predominant cause of HCC. These projections highlight the shifting landscape of HCC etiology (100–102).

Hepatocellular carcinoma (HCC) is the sixth most prevalent cancer globally and the third leading cause of cancer-related deaths (1). In China, the 5-year relative survival rate for HCC is below 30% (2). Furthermore, due to the rising prevalence of risk factors such as metabolic dysfunction-associated steatotic liver disease (MASLD) and alcohol consumption, geographic regions that previously had low incidence rates are now experiencing an increase in HCC cases (**Figure 1**) (1). **Figure 1b** illustrates the evolving etiological landscape of HCC across global, European, and East Asian populations from 2014 to 2034: while the proportion of hepatitis B virus (HBV)- and hepatitis C virus (HCV)-related HCC is projected to decline, MASLD and alcohol-associated liver disease (ALD)–related HCC is anticipated to increase progressively, particularly in Europe; conversely, HBV remains the dominant etiological driver in East Asia. These trends reflect changing HCC epidemiology and call for increased attention. Surgical resection or liver transplantation remains the most effective treatment for patients with early-stage HCC (3). Although both approaches offer a 5-year overall survival (OS) rate exceeding 70% (4, 5), the recurrence rate following surgical resection is as high as 80%, while liver transplantation faces critical constraints including donor shortage and prohibitive costs (4), which significantly limits its therapeutic efficacy. For advanced-stage HCC patients, systemic therapies serve as the mainstay of treatment, though their survival benefits appear to have reached a plateau with limited prospects for dramatic improvement (6). Therefore, perioperative interventions aimed at reducing postoperative recurrence and improve resectability are critically important.

Neoadjuvant therapy refers to the use of systemic or local treatments prior to the definitive treatment. This strategy has been extensively applied in the management of various solid tumors, such as breast cancer, colorectal cancer, and lung cancer (7–10), demonstrating favorable clinical outcomes. The primary aim of neoadjuvant therapy is to downstage the tumor, thereby increasing the rate of R0 (complete resection with no microscopic residual tumor) resection and reducing the risk of postoperative recurrence. In the specific context of resectable HCC, neoadjuvant therapy focuses on eradicating micrometastatic disease and improving long-term outcomes. micrometastasis constitutes a key risk factor for recurrence (11), where neoadjuvant therapy allows early intervention in micrometastatic lesions, thus improving treatment efficacy (12). Furthermore, neoadjuvant therapy provides advantages such as assessing treatment responsiveness and minimizing delays in therapy caused by postoperative complications (13).

The evolution of neoadjuvant therapy for HCC reflects a profound transformation in comprehensive liver cancer treatment strategies. The early stage primarily explored cytotoxic chemotherapy, single-agent targeted drugs, and local interventional therapies (14). However, clinical benefits were significantly limited by insufficient objective response rates and inefficient tumor downstaging. With the introduction of immune checkpoint inhibitors, the treatment paradigm underwent a fundamental shift: dual immune checkpoint blockade strategies and targeted-immunotherapy combination regimens achieved breakthrough improvements in major pathological response rates (15). The integration of local therapies with systemic immunotherapy enhanced systemic antitumor responses through antigen release effects, further expanding the therapeutic repertoire for resectable HCC with high-risk features (16).

Given that neoadjuvant systemic therapy for resectable HCC remains in a phase of rapid evolution, clinical guidelines show variations in both recommendation strength and specific protocols, primarily relying on expert consensus and preliminary clinical evidence rather than established standard-of-care. Current international guidelines have not established a standardized pathway for neoadjuvant systemic therapy in this setting. The American Association for the Study of Liver Diseases (AASLD) guidelines explicitly recommend against routine systemic therapy for patients scheduled for hepatic resection (17), while the National Comprehensive Cancer Network (NCCN) and European Society for Medical Oncology (ESMO) guidelines adopt a more cautious and exploratory stance, acknowledging the potential value of neoadjuvant approaches primarily within clinical trial contexts (18, 19). These divergent positions reflect the absence of mature phase 3 data specifically in resectable HCC and the inherent tension between the promise of perioperative systemic therapy and the need to avoid compromising surgical candidacy through treatment-related toxicity or disease progression (17–20). Notably, the Chinese Expert Consensus on Neoadjuvant and Conversion Therapy for Hepatocellular Carcinoma delineates a more refined algorithm: precise clinical staging and risk stratification are used to assign specific locoregional or systemic regimens—including exact drug combinations and radiotherapy doses—to distinct patient subgroups (**Table 1**) (21). This consensus represents the most detailed guideline-level guidance for neoadjuvant therapy in resectable HCC to date, though it remains grounded in expert opinion rather than prospective randomized evidence.

**Table 1:** Recommended neoadjuvant and conversion therapies for HCC.

Applicable Conditions	Recommended Regimens
CNLC Stage IIa	lenvatinib / lenvatinib + camrelizumab / TACE + lenvatinib + camrelizumab / MDT
CNLC Stage IIb	TACE or combined sintilimab
CNLC Stage IIa~IIIa	TACE / TACE-MWA / TARE
CNLC Stage IIa~IIb + exceeding Milan criteria (BCLC Stage A/B)	FOLFOX-HAIC
CNLC Stage Ia~IIIb + insufficient FLR	ALPPS / PVE
CNLC Stage IIIa + Vp1-2/Vv1 tumor thrombus	Concurrent boost radiotherapy
CNLC Stage Ia~IIb + Child-Pugh A/B CNLC Stage IIIa + Vp3~Vp4/Vv2~Vv3 tumor thrombus CNLC Stage IIIb + pulmonary oligometastases/extrahepatic lymph node metastases	Concurrent boost radiotherapy + combined with systematic therapy

This table summarizes treatment recommendations from the Chinese Expert Consensus on neoadjuvant and conversion therapies for HCC (23). Therapies are categorized according to China Liver Cancer (CNLC) staging criteria and specific clinical conditions. (Abbreviations defined in the Abbreviations section).

Nevertheless, none of the guidelines specify the optimal treatment duration, the mandatory drug-free interval before surgery, or the pathologic or radiologic criteria that define “success.” The absence of these pivotal decision anchors—coupled with recommendations derived largely from retrospective cohorts or heterogeneous meta-analyses—highlights the current lack of systematic precision. Close monitoring of ongoing clinical trials is therefore imperative to resolve these critical knowledge gaps. This review aims to critically evaluate the current landscape and clinical applications of neoadjuvant therapy in HCC. We synthesized the latest and the cutting-edge advancements in the field and highlighted innovative strategies in patient selection. Furthermore, this review offers an overview of ongoing clinical trials, highlighting their potential impact on the future landscape of neoadjuvant treatment for HCC.

## 2 Neoadjuvant systematic therapy

Neoadjuvant systemic therapy for HCC has evolved through successive phases: from the limited efficacy of the chemotherapy era, through the disappointing results of the targeted therapy era, to the breakthrough progress of the immunotherapy era. Early chemotherapy regimens failed to achieve clinical promotion due to low objective response rates and compounded hepatotoxicity. Although targeted agents such as sorafenib demonstrated tumor shrinkage potential in individual cases, the objective response rate with monotherapy was only 2–18%, and poor tolerability limited their neoadjuvant applications. The emergence of immune checkpoint inhibitors (immune checkpoint inhibitor (ICI)s) represented a turning point, with monotherapy achieving a pathological complete response rate of 22%. Currently, immunotherapy combined with antiangiogenic agents represents the most promising direction, with combination regimens demonstrating objective response rates as high as 83.3% and pathological complete response rates of 26.1% in resectable HCC, providing new opportunities to address the clinical challenge of high post-operative recurrence rates.

### 2.1 Neoadjuvant targeted therapy

Current targeted therapies for HCC predominantly comprise two classes: (1) tyrosine kinase inhibitors (TKI, such as sorafenib, lenvatinib, donafenib) and (2) vascular endothelial growth factor (VEGF) pathway inhibitors (bevacizumab). However, the available evidence is derived predominantly from small, single-arm phase II trials, limiting both reliability and generalizability. Sorafenib, a multikinase inhibitor, exerts dual antiproliferative effects by targeting Ser/Thr kinase Raf to suppress tumor cell proliferation and inhibiting VEGF receptor 2/platelet-derived growth factor receptor (VEGFR2/PDGFR) (Platelet-derived growth factor receptor) to block endothelial cell growth (22).

Emerging clinical evidence from phase II/III trials has established sorafenib’s efficacy in the adjuvant setting for advanced HCC, providing a rationale for exploring its neoadjuvant applications. A multicenter phase II trial (NCT 01182272) demonstrated disease stabilization in all resectable HCC patients receiving neoadjuvant sorafenib, with all patients proceeding to surgery and 88% (22/25) attaining R0 resection (23).

Yokoo H. et al. evaluated the efficacy of pre-operative lenvatinib followed by hepatectomy for huge (≥10 cm) resectable hepatocellular carcinoma in a single-center retrospective study. Thirty patients were enrolled: nine received neoadjuvant lenvatinib before surgery (LEN group) and 21 underwent upfront surgery (UFS group). The 3-year recurrence-free survival rate was significantly higher in the LEN group (66.7% *vs* 16.1%; P = 0.027), and distant metastasis was less frequent (22.2% *vs* 47.6%). Pathological examination revealed extensive (>70%) tumor necrosis induced by lenvatinib (24). Furthermore, high expression of VEGF and/or FGF in tumor tissue correlates with better objective response to lenvatinib monotherapy, while elevated serum FGF-21 levels may indicate longer overall survival in treated patients. These findings suggest that baseline angiogenic profiles may inform patient selection, yet their integration into routine preoperative decision-making remains preliminary given the small sample sizes and single-center designs of the deriving studies.

The evident appeal of neoadjuvant targeted therapy lies in its oral administration, established safety profile in the metastatic setting, and capacity to induce rapid tumor devascularization—attributes that theoretically minimize surgical delay and perioperative risk. tyrosine kinase inhibitors (TKIs) also hold mechanistic promise for “normalizing” tumor vasculature, potentially improving chemotherapy delivery when combined with other systemic or locoregional modalities (25). However, the limitations are substantial and often underappreciated in the enthusiasm surrounding positive phase II signals. The evidence base consists almost entirely of small, uncontrolled series in which observed benefits cannot be disentangled from inherent patient selection: individuals who tolerate neoadjuvant therapy long enough to reach surgery may represent a fundamentally fitter cohort with less aggressive tumor biology. The critical, unresolved tension centers on the risk–benefit calculus of surgical delay. For resectable disease, does the potential survival gain from preoperative cytoreduction outweigh the risks of disease progression during TKI therapy and the hepatic/systemic toxicities that may compromise postoperative recovery? Sorafenib-induced hand–foot skin reaction, hypertension, and diarrhea are manageable in the palliative setting but assume greater significance when they threaten to derail a potentially curative operation. Additionally, the optimal duration of neoadjuvant TKI administration remains undefined—too brief may yield insufficient tumor response, while prolonged administration risks unnecessary toxicity and the theoretical concern of promoting resistant clones. These uncertainties underscore that neoadjuvant targeted therapy should currently be regarded as a highly exploratory, individualized strategy rather than a standard intervention.

### 2.2 Neoadjuvant immunotherapy

Immunotherapy has revolutionized HCC management, bringing neoadjuvant approaches to the forefront of research (**Table 2**). Preoperative ICIs may prime *de novo* T cell-mediated immunity by preserving tertiary lymphoid structures (TLS), expanding preexisting tumor-specific T cells, and diversifying T-cell repertoires, potentially offer enhanced efficacy compared to adjuvant therapy (26). These tumor-specific memory T cells exhibit enhanced capacity to detect micrometastatic foci, thereby reducing recurrence after curative resection (27–29).

**Table 2:** Efficacy of Neoadjuvant Immunotherapy in HCC.

Lead Author (year) (Ref.)	Regimen	Study Design	Number of Patients	MPR Definition	MPR Rate	pCR Rate
Marron TU et al. (2022) (32)	Cemiplimab monotherapy	Phase II	21	>70% tumor necrosis	20% (4/20)	15% (3/20)
Kaseb AO et al. (2022) (34)	13 Nivolumab ± 14 Ipilimumab	Phase II	27	>60% tumor necrosis	Nivolumab group 33% (3/9) Nivolumab ± Ipilimumab group 28% (3/11)	Nivolumab group 22% (2/9) Nivolumab ± Ipilimumab group 28% (3/11)
LaPelusa M et al. (2024) (35)	Nivolumab + Ipilimumab (Based on the trial of Kaseb AO et al)	Phase II	18	>70% tumor necrosis	33% (6/18) (Unstratified)	–
D'Alessio A et al. (2022) (36)	Nivolumab + Ipilimumab	Phase Ib	17	–	78% (7/9)	22% (2/9)

Abbreviations defined in the Abbreviations section.

The clinical heterogeneity of immunotherapy response in HCC is increasingly linked to baseline molecular and immune biomarkers. Among patients receiving ICI monotherapy, those with activating WNT/β-catenin signaling pathway alterations have demonstrated a significantly lower disease control rate compared with patients without such alterations (0% vs. 53%), positioning WNT/β-catenin activation as a candidate negative predictive biomarker (30). However, the reliability of this association remains contentious; its predictive value may be influenced by tumor heterogeneity, co-mutation background, and detection methodology, and it has not proven consistent across all patient cohorts. High expression of T-effector cell-related genes such as GZMB, PRF1, and CXCL9 or specific inflammatory gene signatures including CD274 and CD8A has been associated with better response and survival benefits following anti-programmed cell death-1 (PD-1)-based therapies (31). It should be noted that these gene signatures, largely derived from bulk tumor sequencing, inadequately reflect intratumoral spatial heterogeneity—a limitation that constrains their broader clinical utility.

Cemiplimab, a high-affinity PD-1 inhibitor, demonstrates promising antitumor activity in patients with resectable HCC. In a phase II trial conducted by Marron et al., 21 resectable HCC patients received two cycles of neoadjuvant cemiplimab, followed by eight cycles of adjuvant therapy after resection. Among the 20 patients who underwent resection, 20% achieved major pathological necrosis (>70%), 15% partial response (PR), and 65% stable disease (SD). Tumors with ≥50% necrosis exhibited higher densities of tumor-infiltrating lymphocytes (tumor-infiltrating lymphocyte (TIL)s), suggesting that baseline immune infiltration may serve as a predictive marker for treatment response (32). This observation aligns with broader findings that the pre-treatment tumor immune microenvironment status holds significant predictive value across multiple neoadjuvant ICI regimens.

Antibodies targeting programmed cell death-1 (PD-1) and cytotoxic T-lymphocyte-associated protein-4 (CTLA-4) have become the backbone of systemic therapy for multiple advanced or metastatic malignancies. CTLA-4 blockade acts at the lymph-node priming phase by disrupting B7–CD28 competitive inhibition, thereby expanding the peripheral T-cell clonal repertoire. PD-1 blockade operates within the tumor microenvironment to release PD-1/programmed death-ligand 1 (PD-L1)-mediated exhaustion signals and restore cytotoxic function. Dual inhibition concurrently up-regulates the PI3K–AKT–mTOR axis and IL-2/IFN-γ secretion, reduces regulatory T-cell (Treg) infiltration, and increases the Teff/Treg ratio, producing non-redundant, cascade-amplified, synergistic antitumor activity (33).

In the randomized phase II trial conducted by Kaseb et al., 27 patients with resectable HCC were treated with either perioperative nivolumab monotherapy (240 mg every 2 weeks preoperatively and 480 mg every 4 weeks postoperatively) or a combination of nivolumab (480 mg every 4 weeks) and ipilimumab (1 mg/kg every 6 weeks). Major pathological response (MPR, defined as ≥60% tumor necrosis) was reached in 33% and 27% of patients in the monotherapy and combination groups, respectively. Remarkably, none of patients who achieved MPR experienced disease recurrence during a median follow-up of 24.6 months, whereas 50% of non-MPR patients relapsed. Additionally, the median progression-free survival (PFS) differed significantly between the monotherapy and combination groups, at 9.4 and 19.5 months (hazard ratio (HR), Hazard Ratio=0.89), respectively (34). This study provides the first clinical evidence supporting the safety and preliminary efficacy of neoadjuvant immunotherapy in HCC, highlights MPR as a potential surrogate endpoint. Cellular biomarker analysis from this trial revealed that increased CD8+ T-cell infiltration, a higher CD8+ T cell to regulatory T cell ratio (CD8+/Treg) ratio, and expansion of effector T cells (Eomes+CD45RO+) were characteristic features of patients achieving MPR, whereas enrichment of V-domain Ig suppressor of T cell activation (VISTA)+ myeloid cells was associated with non-MPR status. Post-treatment upregulation of intratumoral granzyme B expression directly demonstrated successful activation of effector T-cell cytotoxic function, and its expression level could serve as a dynamic predictive marker for immune activation and treatment efficacy (35). Nevertheless, the immune microenvironment is highly dynamic, and static sampling prior to treatment may fail to fully capture its true predictive capacity.

The phase Ib PRIME-HCC trial (NCT03682276) conducted in 17 patients with early-stage HCC, reported a 78% pathological response rate (22% complete), a 92% disease control rate (dendritic cell (DC)R), and manageable toxicity [73% any-grade treatment-related adverse events; 7% grade 3 alanine aminotransferase/aspartate aminotransferase (ALT/AST) (Alanine Aminotransferase/Aspartate Aminotransferase) elevation] with nivolumab-ipilimumab. Median time-to-surgery remained 2.5 months (36). In a separate single-arm trial enroling 43 patients treated with the identical combination, 34.9% achieved >10% tumor shrinkage. Among the 24 patients who underwent resection, 33.3% attained a MPR (>90% necrosis). Four-year PFS and OS for the entire cohort were 44% and 60%, respectively, whereas the subgroup receiving curative-intent surgery reached 68% and 86%, indicating that neoadjuvant combination therapy followed by resection can confer durable survival benefit (37). The observed rapid tumor shrinkage following immunotherapy underscores its distinct “fast-acting” mechanism compared to the gradual response seen with targeted therapies, suggesting a unique antitumor mode of action driven by immediate immune activation rather than gradual vascular remodeling. This temporal distinction carries practical implications: immunotherapy may be particularly suited for scenarios where rapid cytoreduction is desirable to facilitate timely surgery. However, translation into routine practice remains constrained by several unresolved tensions. Efficacy heterogeneity is marked, with response rates varying from 20% to 78% across trials—a range that reflects differences in patient selection, trial design, and potentially biomarker-enriched populations, but also exposes the lack of reliable pretreatment predictive tools. The clinical relevance of varying thresholds of tumor necrosis as a pathologic response endpoint in HCC requires rigorous validation; while MPR (variously defined as ≥60% or ≥70% necrosis) has been proposed as a surrogate for recurrence-free survival, a globally standardized definition has yet to be established, and the inherent heterogeneity of necrotic regions necessitates standardized pathologic assessment protocols. Furthermore, perioperative immunotherapy introduces unique management complexities, including the need to distinguish immune-related adverse events from surgical complications, the optimal timing of surgery relative to ICI administration, and the unresolved question of whether patients with suboptimal pathological responses should receive intensified adjuvant regimens.

### 2.3 Neoadjuvant immuno-targeted combination therapy

The theoretical rationale for combining targeted therapy with immunotherapy rests on complementary mechanisms that address the limitations of each approach when used alone. TKIs suppress angiogenesis and remodel the immunosuppressive tumor microenvironment (TME), while ICIs reinvigorate antitumor immunity, creating a synergistic “immune-normalizing” effect that may overcome the primary and acquired resistance observed with monotherapy (25). This mechanistic synergy is exemplified in multiple clinical studies (38, 39).

CARES-009 is a randomized phase 2/3 trial conducted in China, the first to assess perioperative immunotherapy combined with anti-angiogenic therapy for resectable hepatocellular carcinoma. The trial enrolled 312 patients with early-stage resectable HCC and randomized them 1:1 to either perioperative neoadjuvant treatment with camrelizumab plus rivoceranib or surgery alone. The results demonstrated that perioperative camrelizumab combined with rivoceranib significantly prolonged event-free survival compared with surgery alone (median 42.1 months vs. 19.4 months; HR 0.59; P = 0.0040), and increased the major pathological response rate (35% vs. 8%). The R0 resection rate was 100% in both groups. Subgroup analyses indicated that patients achieving MPR (tumor necrosis >90%) had the most favorable prognosis, supporting the use of pathological response as a surrogate endpoint for treatment strategies. However, the combination therapy group experienced a higher rate of grade 3 or higher adverse events (38% vs. 0%), and two treatment-related deaths occurred during the neoadjuvant treatment period. Approximately 90% of the enrolled population had concomitant hepatitis B virus (HBV) infection, providing new evidence for perioperative treatment of HBV-related HCC. However, the follow-up duration was relatively short, and mature overall survival data are not yet available (40).

In an exploratory study of neoadjuvant treatment for resectable HCC, Xia et al. conducted a single-arm, open-label phase II clinical trial to evaluate the efficacy of camrelizumab (a PD-1 inhibitor) combined with apatinib (a VEGFR2 inhibitor) as a perioperative regimen. The study enrolled 18 patients with resectable HCC who received 3 cycles of combination neoadjuvant therapy, followed by surgical resection and 8 cycles of adjuvant treatment. The clinical results demonstrated that the objective response rate (ORR) was 16.7% and 33.3% according to Response Evaluation Criteria in Solid Tumors (RECIST) v1.1 and modified Response Evaluation Criteria in Solid Tumors (mRECIST) criteria, respectively. Among the 17 patients who underwent surgery, the major pathological response (MPR) rate was 17.6% and the complete pathological response (pCR) rate was 5.9%, while the 1-year recurrence-free survival (RFS) rate reached 53.85%. The unique academic value of this study lies in its utilization of a multidimensional omics platform to comprehensively elucidate key biomarkers predictive of treatment response and postoperative recurrence. Regarding the tumor immune microenvironment, treatment responders demonstrated significantly higher baseline infiltration of dendritic cells (DCs) in tumor tissue, accompanied by elevated expression of a series of immune activation-related genes, suggesting that high DC infiltration may serve as a potential immune marker to predict treatment efficacy and lower recurrence risk with this regimen. Regarding peripheral blood biomarkers, patients with higher baseline circulating tumor DNA (ctDNA) mutation burden were more likely to achieve pathological response, and those who achieved dynamic ctDNA clearance during the postoperative adjuvant treatment period demonstrated significantly prolonged recurrence-free survival, demonstrating promising applications of ctDNA as a tool for monitoring minimal residual disease (MRD). Furthermore, through proteomics analysis, this study explored the resistant heterogeneity among multiple lesions and found significant differences in the expression of glycolipid metabolism pathway-related proteins (such as FASN, PKM, and TPI1) between responder and non-responder lesions, indicating that intratumoral metabolic heterogeneity may be an important molecular mechanism underlying the differential drug resistance among different lesions. This provides a novel perspective for clinical evaluation of neoadjuvant efficacy in multifocal HCC (41).

In borderline resectable HCC, Ho et al. investigated cabozantinib plus nivolumab as neoadjuvant therapy in 15 patients. Among them, 80% achieved R0 resection, with 5/12 (42%) patients exhibiting a MPR (≥90% tumor necrosis). All responders remained disease-free for over 7.6 months (42). Beyond these clinical endpoints, spatial proximity between B cells and macrophages emerged as a cellular spatial-relationship biomarker: in tumor samples from responders, B cells and T cells were spatially closer to CT11 macrophages (expressing lower levels of Arg1 and higher levels of PD-L1) and farther from CT10 macrophages (expressing higher levels of Arg1). Spatial transcriptomic analysis of cabozantinib combined with nivolumab therapy revealed that responders exhibited upregulation of PAX5 activation and B-cell maturation-related genes (CD19/CD79A), whereas non-responders showed activation of tumor proliferation pathways such as E2 transcription factor/MYelocytomatosis oncogene (E2F/MYC) (43). This represents a promising direction toward spatially resolved biomarkers. However, such spatial profiling technologies are complex, costly, and currently challenging to implement in routine clinical pathology. The clinical applicability and generalizability of these spatial biomarkers remain to be validated.

The strengths of immuno-targeted combination therapy are considerable. By simultaneously targeting angiogenic and immune escape pathways, combination regimens achieve higher pathological response rates than either modality alone, and the durability of response appears superior—suggesting that systemic immune activation may more effectively eliminate micrometastatic foci than purely antiproliferative treatment. The CARES-009 data, in particular, represent the highest level of evidence currently available for neoadjuvant systemic therapy in HCC, with a phase III design providing robust support for event-free survival benefit.

Yet the shortcomings are equally prominent and warrant careful scrutiny. Combination therapy cumulatively incorporates the toxicities of both drug classes. In the CARES-009 study, the rate of grade 3 or higher treatment-related adverse events was 38%, including treatment-related deaths (40). For patients with resectable disease, any severe toxicity may offset potential benefits or even render patients ineligible for curative surgery—a risk that assumes particular gravity given that the comparator in CARES-009 was surgery alone, not an alternative systemic regimen. The majority of positive data originate from rigorously selected patient populations; whether HCC patients with comorbidities such as decompensated cirrhosis, poor liver function reserve, or autoimmune tendencies can tolerate combination therapy remains unknown. Furthermore, fundamental strategic questions lack clear answers. The optimal treatment duration, timing of efficacy assessment, and criteria for proceeding to surgery versus switching to alternative regimens are undefined. For patients with borderline resectable disease, does the primary goal of combination therapy center on achieving sufficient tumor shrinkage to enable R0 resection, or on generating systemic immune memory to prevent postoperative recurrence? The former objective may favor more potent TKI-based combinations with shorter treatment windows, whereas the latter may prioritize ICI-heavy regimens with longer neoadjuvant courses—yet current evidence offers little guidance on how to operationalize this distinction in clinical practice. Compared with monotherapy, the mechanistic complexity of combination regimens makes these questions substantially more difficult to answer, as the contribution of each component to both efficacy and toxicity becomes harder to isolate.

## 3 Neoadjuvant interventional therapy

Currently, neoadjuvant transarterial chemoembolization (TACE) is widely used in the treatment of resectable HCC, but its efficacy remains controversial due to inconsistent therapeutic benefits and potential complications. Amisaki et al., in a single-center retrospective study including 68 resectable HCC patients, compared long-term outcomes between the preoperative TACE group (n = 34) and surgery-alone group (n = 34), demonstrating that OS and RFS in the TACE group were significantly inferior to the control group (OS: P = 0.014; RFS: P = 0.043), with a higher rate of early recurrence (P = 0.035). These detrimental effects may stem from TACE-induced hypoxia-driven upregulation of VEGF and hypoxia-inducible factor-1α (HIF-1α) promoting angiogenesis and metastasis, as well as selective elimination of well-differentiated tumor cells that leave behind more aggressive, poorly differentiated clones (44). In contrast, Giannone et al. conducted a multicenter retrospective propensity score-matched (PSM) study across 9 high-volume European centers involving 384 resectable large HCC patients (>5 cm), observing that although overall OS and disease-free survival (DFS) showed no significant difference before and after matching (OS: P = 0.172; DFS: P = 0.935), subgroup analyses revealed that patients with tumor diameter ≥10 cm (P = 0.045), solitary lesions (P = 0.052), and those requiring portal vein embolization/ligation (P = 0.087) might benefit from preoperative TACE, suggesting that tumor burden and hepatic parenchymal reserve requirements may be important clinical factors for patient selection (45). Fang et al. further provided high-level evidence through a prospective randomized controlled trial (ChiCTR2200055618), in which 164 resectable HCC patients were randomly assigned to the neoadjuvant TACE plus surgery group (n = 82) or surgery-alone group (n = 82). Results demonstrated that OS rates at 1/2/3 years (97.2%/88.4%/71.6%) and PFS in the TACE group were significantly superior to the surgery-alone group (82.4%/60.4%/45.7%, P = 0.0011), with good safety profile (46). Notably, none of the above three studies reported postoperative pathological response rates or tumor necrosis extent, and the absence of these data limits deeper understanding of the mechanisms underlying this strategy’s benefits and the establishment of precise patient selection models. These apparently contradictory findings are not merely statistical noise—they reflect genuine biological heterogeneity in TACE response that is governed by tumor burden and underlying liver biology. For early-stage, low-burden tumors (solitary lesions ≤5 cm, or up to three lesions each ≤3 cm, or Barcelona Clinic Liver Cancer (BCLC) stage A), TACE may not reduce recurrence and could even compromise long-term survival by inducing a hypoxic tumor microenvironment that promotes the growth of residual, more aggressive cancer cells. In this context, the ischemic and inflammatory sequelae of embolization may outweigh its cytoreductive benefits, particularly in patients with favorable baseline prognostic features for whom upfront surgery already offers excellent outcomes. Conversely, for patients with large, solitary tumors or those requiring portal vein modulation to enable safe resection, preoperative TACE may demonstrate survival benefits by reducing intraoperative tumor burden and facilitating more complete resection. This dichotomy suggests that the net benefit of neoadjuvant TACE is critically dependent on patient selection and tumor biology—a nuanced picture that is often lost in binary “effective versus ineffective” summaries.

Studies have revealed that TACE-induced hypoxic microenvironments may upregulate VEGF and programmed death-ligand 1 (PD-L1) expression, promoting tumor immune evasion and angiogenesis, thereby restricting long-term survival benefits (47). Consequently, integrating systemic therapies with interventional therapies has emerged as a critical strategy for optimizing neoadjuvant approaches. Through synergistic effects, locoregional therapies promote tumor antigen release, while systemic treatments prevent systemic recurrence and metastasis.

Zhu et al. also applied PD-1 inhibitors (camrelizumab/sintilimab) plus TACE in patients with China Liver Cancer (CNLC) stage II HCC, achieving an ORR of 75%, successful downstaging to resectable CNLC stage I in 70% of cases, with a 1-year DFS rate of 86.6% (47). This study preliminarily validated the potential of TACE combined with PD-1 inhibitors for downstaging in neoadjuvant therapy; however, its limitations—including single-center design, small sample size (n = 20), consecutive enrollment pattern, lack of control group, and absence of postoperative pathological response data—render this conclusion merely an exploratory signal, making it difficult to assess the true added value of the combination strategy compared with TACE monotherapy or surgery alone.

Zhao et al. pioneered the use of Drug-eluting bead TACE (DEB-TACE) combined with tislelizumab in 41 resectable HCC patients, achieving a pathological complete response (pCR) rate of 31.7%, a microvascular invasion (MVI) incidence of 4.9% compared with 60.9% historically, an ORR of 75.0%, and a disease control rate of 100.0%. The 1- and 2-year overall survival rates reached 100.0% and 76.4%, respectively, with a 1-year recurrence-free survival rate of 86.6% (48). DEB-TACE can enhance locoregional control through sustained doxorubicin release and polarization of tumor-associated macrophages toward an M1 phenotype, thereby synergizing with PD-1 blockade. This study was the first to report neoadjuvant pathological response data for TACE combined with immunotherapy in resectable HCC; however, its single-center origin and the lack of explicit clarification regarding pathological response assessment criteria (such as the specific definitions and assessment methods for pCR/MPR) in the manuscript limit its methodological rigor and result reproducibility.

**Figure 2:**
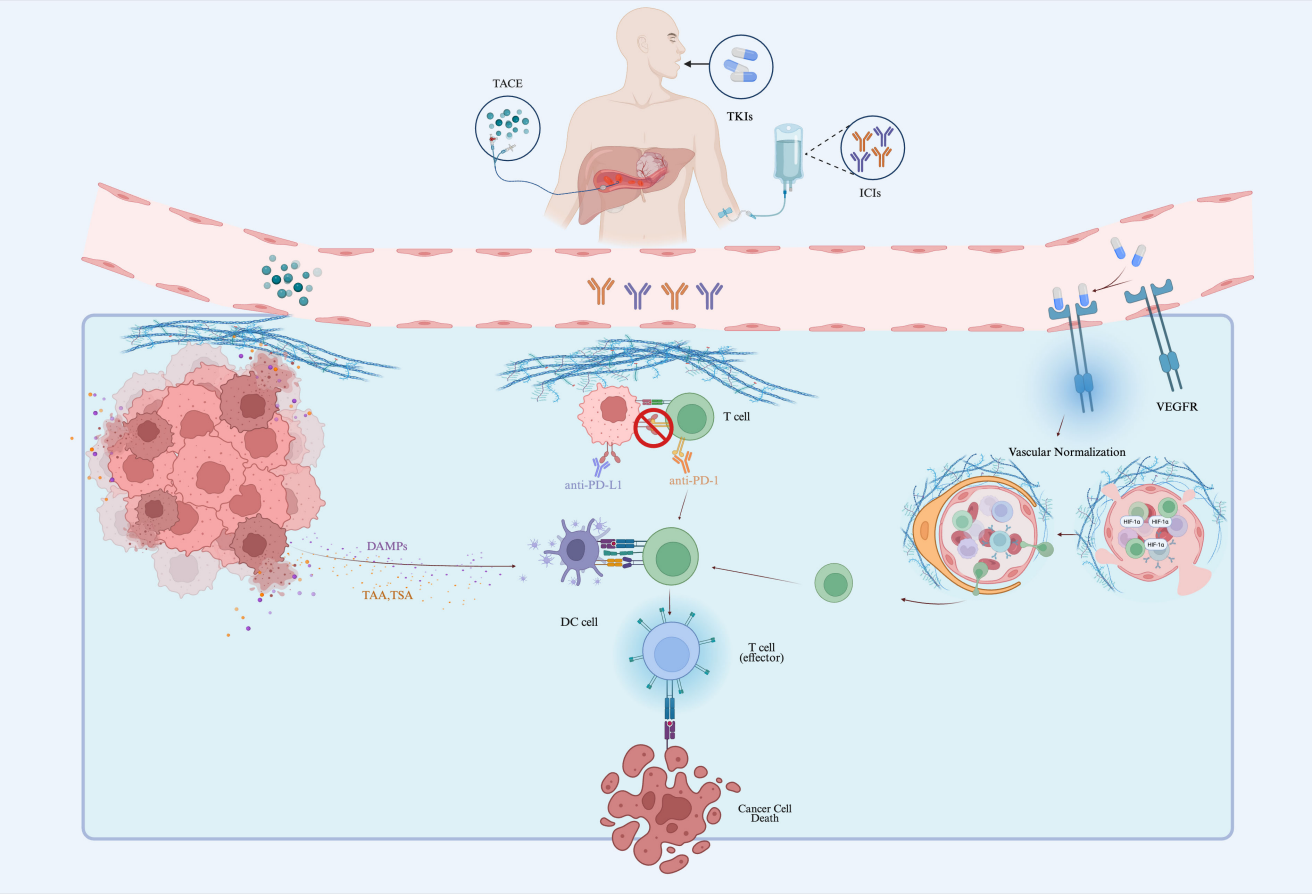
Synergistic Mechanism of TACE, TKIs, and ICIs in Tumor Treatment. TACE induces tumor necrosis, releasing tumor-associated antigens (TAAs) and damage-associated molecular patterns (DAMPs) that activate dendritic cells (DCs) in the tumor microenvironment. Activated DCs migrate to lymphoid organs to prime T-cell responses, while concurrently, immune checkpoint Inhibitors (ICIs: anti-PD-1/anti-PD-L1 antibodies) block inhibitory checkpoint signals on T cells, reversing exhaustion and promoting effector T-cell expansion. TKIs inhibit vascular endothelial growth factor receptor (VEGFR) signaling, normalizing aberrant tumor vasculature to reduce interstitial pressure and hypoxia, thereby facilitating T-cell infiltration into tumor cores. The concerted action of these three modalities remodels the immunosuppressive landscape, leading to potentiated cytotoxic T-cell activity, significant tumor regression, and downstaging of initially unresectable lesions to operable status. Key cellular interactions include DC-mediated antigen presentation, checkpoint blockade-induced T-cell reactivation, and vascular normalization-driven immune cell trafficking, collectively enabling synergistic tumor control.

Based on the success of combination therapies in neoadjuvant and conversion settings, various combination regimens have also emerged for managing HCC. TACE induces tumor antigen release, TKIs inhibit hypoxia-driven VEGF-mediated angiogenesis, and ICIs reverse T-cell exhaustion (**Figure 2**) (49). A Chinese multicenter retrospective study compared TACE combined with targeted therapy versus TACE combined with targeted therapy and immunotherapy in patients with resectable HCC. In the triple-therapy group, both median PFS and OS were not reached, whereas in the double-therapy group they were 24.2 months and 30.7 months, respectively. The risks of disease progression and death were both reduced by approximately 55% (P < 0.05). The 1-year OS and PFS rates were 87.5% and 81.3%, respectively. Further analysis indicated that pathological response is a key prognostic factor, with patients achieving MPR or pCR demonstrating significantly better OS and PFS (50).

Despite these encouraging signals, several critical limitations temper enthusiasm for routine adoption. The current research paradigm generally lacks in-depth analysis of “non-responders”—patients who received intensive triple therapy but did not achieve MPR represent a particularly understudied population whose poor outcomes highlight the need for predictive biomarkers to guide treatment selection. Furthermore, the evidence base consists predominantly of retrospective studies and small phase II trials from single centers, with inherent selection biases regarding treatment timing and assessment processes.

Hepatic arterial infusion chemotherapy (HAIC), a locoregional approach, has emerged as an alternative neoadjuvant liver-directed therapy. Unlike TACE, HAIC delivers sustained high-dose chemotherapy without the ischemic sequelae caused by embolization and may exert more favorable effects on the TME (51).

A multicenter retrospective cohort study showed that for resectable solitary large HCC (tumor diameter ≥7 cm), neoadjuvant hepatic arterial infusion with the FOLFOX regimen (HAIC) significantly improved patient survival. The study included 397 patients, with 129 receiving neoadjuvant HAIC followed by surgery and 268 undergoing surgery alone. In the intention-to-treat analysis, the neoadjuvant HAIC group demonstrated significantly higher 1-year, 3-year, and 5-year overall survival rates (97.5%, 80.7%, 64.7%) and disease-free survival rates (71.5%, 61.7%, 59.5%) compared to the surgery-only group (83.3%, 62.9%, 53.8% and 48.8%, 32.5%, 26.2%, respectively), with hazard ratios for death and recurrence of 0.506 and 0.466, respectively (52). The neoadjuvant HAIC group demonstrated a significantly lower rate of postoperative liver failure compared to the surgery-alone group [8.4% (11/131) vs. 21.4% (28/131), P = 0.005], suggesting that HAIC has a unique advantage in preserving liver function. However, due to the retrospective design of this study, the timing of surgery was determined by individual clinicians rather than standardized criteria, leading to systemic differences in the surgical windows between the neoadjuvant and upfront surgery groups. Hu et al. extended these findings to patients with portal vein tumor thrombosis (PVTT), reporting a 5-year OS rate of 66.4% with neoadjuvant HAIC versus 37.2% in the surgery-only control (*p* < 0.001), with pronounced survival benefits in Vp3/4 (Venous portal tumor thrombosis of segmental branch/Venous portal tumor thrombosis of main trunk) PVTT subtypes (5-year OS: 66.1% *vs*. 22.4%), suggesting that HAIC may enhance surgical radicality by reducing thrombus burden (53). Wei et al. reported the final results of a multicenter phase III trial comparing neoadjuvant FOLFOX-HAIC with surgery alone in patients with resectable BCLC stage A/B HCC beyond the Milan criteria. The HAIC group achieved significantly improved 3-year OS (58.7% *vs*. 42.3%, *p* = 0.002) and median PFS (17.4 *vs*. 9.8 months, *p* < 0.001), with comparable surgical complication rates (*p* = 0.265) (54). In this study, a logistic regression model based on alpha-fetoprotein (AFP) and CRP was constructed for the first time to predict the response to neoadjuvant HAIC (area under the curve (AUC)=0.756), providing a convenient and practical tool for clinical screening of patients who may potentially benefit. However, the number of HAIC cycles (1 to 4 cycles) and the timing of surgery in this study were determined by a multidisciplinary team (MDT) based on individual conditions, leading to insufficient homogeneity in the treatment protocols. Furthermore, the sample size distribution between the Vp1/2 and Vp3/4 types in the PVTT classification was severely unbalanced (with only 6 cases of Vp1), resulting in limited statistical power to draw conclusions regarding the lack of clinical benefit for the Vp1/2 subgroup.

The relative merits of HAIC versus TACE as neoadjuvant locoregional therapy warrant careful comparative consideration. HAIC offers the advantage of delivering high-dose chemotherapy without inducing the hypoxic microenvironment that may paradoxically stimulate tumor progression, as has been observed with TACE. The phase III data from Wei et al. provide the highest level of evidence currently available for neoadjuvant locoregional therapy in resectable HCC, establishing HAIC as a benchmark against which other approaches should be measured. HAIC may be particularly advantageous in scenarios involving PVTT, where the absence of embolization-related ischemia reduces the risk of further compromising portal venous circulation. Conversely, TACE offers the practical benefit of widespread availability, established reimbursement pathways in many healthcare systems, and the potential for combining with systemic therapy through mechanisms such as DEB-TACE-mediated macrophage polarization. The choice between these modalities should ideally be informed by tumor characteristics (size, vascular involvement, liver function reserve) and institutional expertise, yet the absence of high-level head-to-head comparative evidence between HAIC and TACE in the same indication population leaves clinical decision-making without a solid evidence base. Furthermore, the optimal perfusion regimen for HAIC has not been standardized, and its hematological toxicity and impact on liver function require evaluation in broader populations, including those with borderline hepatic reserve.

Among locoregional therapies for HCC, ablation techniques have secured a distinct position in perioperative protocols due to their minimally invasive nature and high target specificity. Notably, post-ablation pathological responses, particularly complete necrosis rates, have been validated as critical predictors of post-treatment recurrence and survival, informing personalized treatment planning (55). Post-embolization tumor hypoxia and dehydration improve heat conduction, which may weaken the “heat-sink effect” associated with radiofrequency ablation (RFA), potentially narrowing its performance gap with microwave ablation (MWA). Simultaneously, since TACE itself causes partial necrosis, both energy platforms can achieve equally thorough ablation outcomes in combined treatment scenarios (56).

Additionally, ablation not only serves as a neoadjuvant modality for surgery or transplantation but also functions as a radical treatment method itself. Specifically, prior to planned curative ablation, combining it with other treatment modalities can overcome the technical limitations of ablation, clear micrometastases, and improve long-term outcomes (57, 58). In a multicenter retrospective analysis of 468 patients with recurrent HCC and microvascular invasion (MVI), Peng et al. found that neoadjuvant conventional TACE (cTACE) combined with RFA significantly improved 5-year OS (42.5% *vs* 28.7%) and RFS (24.4% *vs* 9.3%) compared to RFA alone, with consistent efficacy even in recurrent lesions ≤3 cm (HR = 0.52), suggesting cTACE may enhance ablation completeness through facilitating micrometastasis clearance and mitigating heat sink effect (59). Supporting the efficacy of combination approaches, a randomized trial by Oyama et al. demonstrated that cisplatin-based HAIC combined with ablation significantly reduced the risk of intrahepatic distant recurrence (HR = 0.468, *p* = 0.022), particularly benefiting early-stage HCC patients with solitary tumors and Child-Pugh class A (HR = 0.479), despite no significant improvement in overall RFS (HR = 0.597, *p* = 0.094) (60). The positioning of ablation within the neoadjuvant paradigm, however, requires careful contextualization. Unlike TACE and HAIC, which are primarily used as standalone neoadjuvant cytoreductive strategies, ablation is more commonly employed in combination regimens or as a definitive treatment for small, early-stage tumors where it may obviate the need for surgery altogether. When used as a true neoadjuvant modality prior to resection, its role is less well defined, and the evidence base is limited by methodological constraints. Inherent biases may exist regarding treatment timing and assessment processes; some patients undergo liver transplantation before scheduled follow-up evaluations, potentially systematically overestimating the pathological complete response rate. Insufficient sensitivity of evaluation tools may introduce additional bias: most current studies rely on CT for imaging assessment, which has limited ability to differentiate necrotic tissue. Theoretically superior MRI is not yet widely adopted, restricting the accuracy of response evaluation. These limitations collectively suggest that the observed efficacy data require validation within more rigorous and standardized frameworks.

## 4 Neoadjuvant radiotherapy

Recent studies have demonstrated the evolving role of radiotherapy (RT) in multimodal management of HCC. For resectable HCC, neoadjuvant RT demonstrates significant potential. In centrally located HCC, where surgical margins are often compromised by proximity to major vascular structures, Wu et al. conducted a phase II trial evaluating neoadjuvant intensity-modulated radiotherapy (IMRT, 50–60 Gy in 25–30 fractions) followed by surgery. The regimen achieved a 5-year OS of 69.1%, compared to 37.2% in historical controls (*p* < 0.05), and a MPR rate of 34.2%, with comparable intraoperative complication rates to surgery alone (34.2% *vs*. 31.6%) (61). Suggesting that neoadjuvant intensity-modulated radiotherapy (IMRT) has clear value in downstaging and local control for centrally located HCC. However, this study had a single-center, single-arm design and lacked a randomized control; thus, the comparison of the 5-year overall survival (OS) with historical controls was limited by differences in the chronological span, baseline patient characteristics, and the evolution of adjuvant treatments. Additionally, since 92.1% of the patients had HBV infection, caution should be exercised when generalizing these findings to non-HBV endemic regions and to populations with HCV or metabolic-associated HCC. In resectable HCC with underlying Child-Pugh A liver cirrhosis, Lemaire et al. prospectively evaluated yttrium-90 selective internal radiation therapy (SIRT) as a neoadjuvant approach. Among the 23 patients treated with SIRT, 53% successfully underwent resection, and MPR (≥50% necrosis) was achieved in 80% (8/10) of resected specimens (62). However, the clinical translational value of this study was severely limited by a high dropout rate: 30% of the intention-to-treat population failed to undergo surgery due to tumor progression or hepatic function deterioration, with an ITT feasibility of only 53%, suggesting that a considerable proportion of patients might lose surgical opportunity due to neoadjuvant SIRT. Therefore, the role of neoadjuvant SIRT in resectable HCC is currently limited to feasibility exploration, and whether it can become a standard neoadjuvant strategy requires further investigation.

Moving to combination strategies in resectable HCC, Zhong et al. reported the first phase Ib trial evaluating neoadjuvant stereotactic body radiotherapy (SBRT) (3×8 Gy) combined with the anti-PD-1 antibody tislelizumab in early resectable HCC, achieving a radiological response rate (RR) of 63.2% per modified response evaluation criteria in solid tumors (mRECIST) and DCR in 100% of patients, with no surgical delays or severe complications observed. Transcriptomic analysis revealed post-treatment upregulation of T cell activation-related genes and enhanced HLA expression, suggesting immunomodulatory synergy between SBRT and PD-1 blockade (63). This radio-immunotherapy approach is mechanistically compeling: RT induces immunogenic cell death, releasing tumor-associated antigens and creating an “*in situ* vaccine” effect that may be amplified by concurrent ICI administration. The low-dose fractionation scheme (3 × 8 Gy) may be particularly suited for immune priming, as preclinical data suggest that moderate fractionation can enhance antigen presentation without causing excessive lymphodepletion. However, the optimal sequencing, dosing, and patient selection for radio-immunotherapy combinations remain undefined, and the long-term durability of responses observed in this small phase I trial requires confirmation.

In HCC patients with PVTT, Wei et al. reported that neoadjuvant 3D conformal radiotherapy (3DCRT; 18 Gy in 6 fractions) significantly improved 24-month OS over surgery alone (27.4% *vs*. 9.4%, P<0.001) (64). Elevated IL-6 levels emerged as a predictor of RT resistance in this cohort, introducing a candidate biomarker that could guide patient selection for neoadjuvant RT in PVTT. The biological rationale is coherent: IL-6-mediated activation of the JAK–STAT3 pathway promotes tumor cell survival, proliferation, and radioresistance, and baseline IL-6 levels may identify tumors with more aggressive biology that are less likely to respond to radiation. Li et al. further explored a triple-modality neoadjuvant regimen combining RT (30 Gy in 10 fractions) with Lenvatinib and sintilimab in 20 patients with PVTT, achieving a MPR rate of 60% and introducing the image-pathology concordance rate (IPCR) as a novel exploratory endpoint (65). This triplet strategy challenges traditional BCLC staging by demonstrating that systemic therapy combined with locoregional RT can render advanced PVTT disease resectable with high pathological response rates. However, persistent challenges include IL-6-mediated radioresistance, which may require targeted radiosensitizers for optimization, and the need for further validation of IPCR as a predictive endpoint in larger cohorts. The complexity of triplet regimens also raises practical concerns regarding cumulative toxicity, particularly in patients with compromised liver function, and the optimal integration of these intensive approaches into existing treatment algorithms remains to be defined.

## 5 Ongoing trials on neoadjuvant therapy for HCC

Neoadjuvant and perioperative strategies for HCC are evolving through innovative approaches that combine locoregional precision interventions with systemic therapies (**Table 3**). Currently, over 15 active clinical trials are investigating diverse neoadjuvant combinations, reflecting a paradigm shift from surgical monotherapy toward multimodal treatment protocols designed to improve resectability, pathological response, and long-term survival outcomes.

**Table 3:** Ongoing clinical trials on neoadjuvant therapy for HCC.

NCT Number	Phase	Study Status	Resectability	Interventions	Primary Outcome Measures	Secondary Outcome Measures	Completion Date
NCT07282184	1/2	Recruiting	Resectable HCC	HAIC + Lenvatinib + PD-1 Inhibitor + surgery *vs* surgery alone	2-Year RFS Rate	TRAE, OS	2028/6/30
NCT07268131	2	Active, Not Recruiting	Resectable HCC exceeding the Milan criteria	TACE+QL1706 (PD-1/CTLA-4 antibody) + surgery	MPR rate	/	2029/10/31
NCT04658147	1	Recruiting	Resectable HCC	Nivolumab + Relatlimab + surgery *vs* Nivolumab + surgery	Surgery Proceed Rate	AE, R0 Resection, pCR, ORR, OS, DFS	2026-06-01
NCT06904014	2	Recruiting	Borderline Resectable HCC	Sintilimab + Lenvatinib + HAIC + surgery *vs* Surgery alone	1-Year DFS Rate	DFS, OS, pCR	2029-06-30
NCT06812039	2	Recruiting	High-risk Recurrence Resectable HCC	HAIC + Sintilimab + Donafenib + surgery *vs* Sintilimab + Donafenib + surgery *vs* Surgery alone	1-year RFS rate	MPR Rate, pCR, ORR, RFS, PFS, OS, TTR	2028-01-31
NCT06492408	2	Recruiting	Resectable HCC	Double ICIs + surgery *vs* Double ICIs + Idarubicin + surgery *vs* Double ICIs + Idarubicin + Bevacizumab + surgery	pCR Rate, MPR Rate	Toxicity, ORR, RFS, OS	2033-12-30
NCT06420440	2	Recruiting	High-risk Recurrence Resectable HCC	HAIC + Lenvatinib + Tislelizumab + surgery *vs* Surgery alone	Median EFS	AE, OS	2027-05-31
NCT06405061	2	Recruiting	Resectable HCC	HAIC + Adebrelimab + Bevacizumab + surgery	ORR	MPR, DCR, R0 Resection, pCR, EFS, OS	2027-08-01
NCT06375486	2	Recruiting	Unresectable HCC	HAIC + Ivonescimab + surgery	ORR	PFS, OS, AE	2026-06-15
NCT06349317	2	Recruiting	Resectable HCC	IMRT + camrelizumab + apatinib + surgery	1-Year EFS Rate	EFS, OS, DFS, R0 Resection, MPR, ORR	2026-06-01
NCT05113186	2	Active, Not Recruiting	High-risk Recurrence Resectable HCC	Percutaneous Ablative + Lenvatinib + surgery	1-Year Local RFS	Response Rate, Recurrence Rate	2026-10-02
NCT04727307	2	Recruiting	Resectable HCC	Radiofrequency Ablation + Atezolizumab + surgery *vs* Radiofrequency Ablation + surgery	RFS		2031-02-01
NCT05613478	3	Recruiting	Resectable HCC	TACE + Camrelizumab + Apatinib + surgery *vs* Camrelizumab + Apatinib + surgery	2-Year EFS	R0 Resection, MPR, pCR, OS, EFS, DFS, AE	2027-11-01
NCT07239245	2	Recruiting	Resectable HCC With High Risk of Recurrence	Atezolizumab + Bevacizumab + TACE + surgery	pCR Rate	MPR, RFS, EFS, OS, AE	2030/11/10
NCT05185505	4	Recruiting	Unresectable HCC Beyond Milan Criteria	TACE + Atezolizumab + Bevacizumab + surgery	Acute Rejection Rate	AE, ORR, Waiting List Removal rate, Transplantation Proceed Rate, Post-treatment Necrosis Rate, RFS, OS, Tumor Biomarkers, Immune Cell Biomarkers	2027-10-31
NCT06741020	1/2	Recruiting	Resectable HCC	HAIC(Adebrelimab + FOLFOX) + surgery	ORR	DCR, TTR, SD, AE	2026-12-30
NCT05908786	1/2	Active, Not Recruiting	Resectable HCC	Atezolizumab + Bevacizumab + surgery *vs* Atezolizumab + Bevacizumab + Tiragolumab + surgery *vs* Tobemstomig + Bevacizumab + surgery	MPR rate	pCR, RFS, EFS, OS, ORR, Downstaging Rate to Milan Criteria, R0 Resection, AE, Surgery Delay Rate	2028-09-30
NCT05171166	2/3	Recruiting	Unresectable HCC	HAIC + TACE + Donafenib + surgery *vs* TACE + Donafenib + surgery	PFS	OS, TTP, ORR, DCR, AE	2027-12-31
NCT07154082	2	Recruiting	Resectable HCC	TACE +Apatinib + Camrelizumab + surgery	Two-year DFS rate	ORR, MPR, TTR, OS, DCR, R0 resection, AE	2028-07-31
NCT07131501	2	Active, Not Recruiting	Resectable HCC	TACE + Iparomlimab + Tuvonralimab + Lenvatinib + Radical surgery	MPR rate	pCR rate, R0 resection, ORR, DCR, EFS, RFS, OS, AE	2029-08-31
NCT07018947	2	Active, Not Recruiting	Resectable Hepatocellular Carcinoma	Atezolizumab + Bevacizumab + surgery *vs* surgery alone	RFS	MPR, pCR, OS, EFS, ORR, R0 resection	2030-11-01
NCT07014150	2	Active, Not Recruiting	Resectable HCC	Iparomlimab + Tuvonralimab + Lenvatinib + surgery	MPR rate	OS, RFS, DCR, objective remission rate, PFS, pCR, AE	2027-05-31
NCT06954116	2	Active, Not Recruiting	Resectable HCC With High Risk of Recurrence	Iparomlimab + Tuvonralimab + Partial hepatectomy	RFS	OS, MPR, RFS, TTR, AE	2029-04-01
NCT06884982	2	Active, Not Recruiting	HCC	QL1706 + Lenvatinib + surgery	MPR rate	CR, R0 resection, EFS	2028-09-30
NCT06664996	2	Recruiting	Resectable HCC	SBRT + Sintilimab + surgery	DFS rate		2026-10-01
NCT06512467	2	Active, Not Recruiting	Resectable HCC	Donafenib + Sintilimab + HAIC + surgery	MPR rate	ORR, DCR, RFS, OS, pCR	2026-12-31
NCT06467799	2	Recruiting	High-risk Recurrent HCC	HAIC + PD-1 + surgery	RFS	ORR, pCR, OS, AE	2027-09-01
NCT06045975	2	Recruiting	HCC	Durvalumab + Tremelimumab + percutaneous ablation	local RFS	Changes of tumorous and non-tumorous perfusion parameters, Changes of size of nodules, Incidences of intra segmental/ extra segmental distant recurrence, OS, AE	2028-09-28
NCT05701488	1	Recruiting	Resectable HCC	Durvalumab + Tremelimumab + surgery *vs* Durvalumab + Tremelimumab + SIRT + surgery	AE	Best Radiologic Response, Best Pathological Response, Median OS, Median PFS, CD8+/CD4+T Cells Level, Dendritic Cells Level, Cytokines Level, Number of Participants with Surgical Complications	2026-10-01
NCT05440864	2	Recruiting	Resectable HCC	Durvalumab + Tremelimumab + surgery	AE	Number of patients experienced surgical delay, ORR, Pathological response rate, R0 resection	2026-11-01
NCT04777942	NA	Recruiting	High-risk BCLC A Stage HCC	TACE-HAIC+Surgery *vs* Surgery alone	PFS	OS	2026-12-30
NCT04615143	2	Recruiting	Resectable RHCC	Tislelizumab + surgery *vs* Tislelizumab + Levatinib + surgery	DFS	ORR, AE, MPR	2027-12-01
NCT04587739	1	Recruiting	HCC	stereotactic body radiation therapy + Surgery	Drop-out rate	Intraoperative number of packed red blood cells transfused, AE, Duration of surgery, Volume of intraoperative blood loss, morbidity rate, mortality rate, Correlation between radiological observations and pathological features, OS, DFS	2029-01-01

Abbreviations defined in the Abbreviations section.

The majority of ongoing trials (NCT07282184, NCT06904014, NCT06420440, NCT06512467, NCT06467799) employ hepatic arterial infusion chemotherapy (HAIC) combined with immune checkpoint inhibitors (ICIs) and tyrosine kinase inhibitors (TKIs), representing the most extensively investigated neoadjuvant strategy. This triple-combination approach is mechanistically rational: HAIC delivers high-concentration cytotoxic agents directly to the tumor vasculature, potentially inducing immunogenic cell death that synergizes with ICIs, while TKIs normalize tumor vasculature and modulate the immunosuppressive microenvironment. For instance, NCT06904014 (Phase 2, n = recruiting) compares sintilimab + lenvatinib + HAIC + surgery versus surgery alone in borderline resectable HCC, with 1-year DFS rate as the primary endpoint and pathological complete response (pCR) as a key secondary measure. Similarly, NCT06420440 targets high-risk recurrence resectable HCC with HAIC + lenvatinib + tislelizumab, prioritizing median event-free survival (EFS) to address the critical unmet need in patients with unfavorable tumor biology.

Several trials explore dual ICI combinations without locoregional therapy, aiming to maximize anti-tumor immune activation. NCT04658147 (Phase 1) investigates nivolumab + relatlimab (CTLA-4 inhibitor) + surgery versus nivolumab monotherapy + surgery in resectable HCC, with surgery proceed rate as the primary outcome—a pragmatic endpoint assessing treatment tolerability and feasibility. Notably, NCT07268131 combines TACE with QL1706, a bispecific PD-1/CTLA-4 antibody, targeting resectable HCC exceeding Milan criteria, with major pathological response (MPR) rate as the primary endpoint. This design reflects the field’s recognition that radiological response may not fully capture the biological efficacy of immunotherapy, necessitating pathological surrogates. NCT06492408 (Phase 2) employs a three-arm design comparing double ICIs + surgery, double ICIs + intratumoral idarubicin + surgery, and double ICIs + intratumoral idarubicin + bevacizumab + surgery, with co-primary endpoints of pCR and MPR rates. This trial uniquely investigates CT-guided intratumoral chemotherapy—direct injection of cytotoxic agents into the tumor microenvironment—hypothesized to enhance localized immunogenic cell death while minimizing systemic toxicity.

Beyond HAIC and TACE, emerging trials incorporate other locoregional modalities to augment systemic immunotherapy. NCT06664996 (Phase 2) combines stereotactic body radiotherapy (SBRT) + sintilimab + surgery, with DFS rate as the primary endpoint. SBRT’s ability to induce abscopal immune effects through radiation-induced antigen release provides biological rationale for synergy with ICIs. NCT06045975 (Phase 2) uniquely investigates durvalumab + tremelimumab + percutaneous ablation in local HCC, with RFS as the primary outcome and detailed imaging biomarkers (changes in tumorous and non-tumorous perfusion parameters) as secondary measures. This trial addresses whether ablative therapies can serve as immune primers in the neoadjuvant context, potentially expanding treatment options for anatomically challenging lesions. Similarly, NCT05701488 (Phase 1) compares dual ICIs + surgery versus dual ICIs + selective internal radiation therapy (SIRT) + surgery, incorporating extensive immune biomarker assessments (CD8^+^/CD4^+^ T cell levels, dendritic cell counts, cytokine profiling), reflecting the field’s shift toward mechanism-driven trial designs. However, only a minority of trials incorporate radiation or ablation, highlighting a significant research gap given the established efficacy of these modalities in bridging and downstaging protocols.

Several important patterns emerge from the current trial landscape. First, pathological endpoints (pCR, MPR) are increasingly prioritized over purely survival-based measures, enabling faster trial readouts and potentially accelerated regulatory pathways. Second, high-risk recurrence populations are explicitly targeted (NCT06812039, NCT06420440, NCT06467799), acknowledging that patients with adverse tumor biology (microvascular invasion, satellite nodules, elevated AFP) derive limited benefit from surgery alone. Third, there is notable emphasis on translational research, with multiple trials incorporating immune profiling, circulating tumor DNA analysis, and radiomics to identify predictive biomarkers.

However, critical challenges remain. The predominance of single-institution trials with relatively homogeneous patient populations (primarily hepatitis B-associated HCC) may limit generalizability to Western cohorts with non-alcoholic steatohepatitis (NASH)- or alcohol-related disease. Additionally, biomarker-driven patient selection remains uncommon; no trials currently stratify enrollment based on PD-L1 expression or tumor mutational burden, despite their established predictive value. The lack of standardized pathological response criteria across trials further complicates cross-trial comparisons. These ongoing trials will clarify whether immunotherapy-based neoadjuvant strategies can meaningfully improve long-term outcomes compared to surgery alone, and whether locoregional therapies provide additive benefit. However, international multicenter collaboration and standardized endpoint definitions are urgently needed to fully realize the potential of neoadjuvant approaches in HCC.

## 6 Biomarkers

The value of neoadjuvant therapy in perioperative management of hepatocellular carcinoma (HCC) has been widely recognized, yet response heterogeneity among patients remains substantial. Identifying reliable predictive biomarkers for treatment efficacy represents a core challenge in achieving precision patient selection. Current evidence has been explored across four dimensions—molecular, cellular, histopathological, and imaging—yet existing research remains predominantly single-center and small-sample in nature, with the clinical translational pathway for biomarkers remaining unclear.

### 6.1 Molecular-level biomarkers

Molecular-level research has revealed two distinct research directions: in the ICI field, baseline gene signatures related to tumor immune activation represent the most consistently identified positive predictive factors across studies; meanwhile, in traditional locoregional or systemic therapy, serum biomarkers and liquid biopsy indicators demonstrate superior clinical operability due to convenient detection and dynamic monitoring capability.

In ICI-related studies, multiple independent investigations point toward highly concordant molecular patterns. Whole-exome sequencing, transcriptomic analysis, and NanoString profiling demonstrate that responder tumors from patients treated with nivolumab plus ipilimumab (37), tislelizumab combined with SBRT (63), cemiplimab (32), and nivolumab monotherapy (34) uniformly exhibit enrichment of IFN-γ signaling pathway, HLA gene expression, and T-cell activation and cytotoxicity-related gene signatures. These concordant findings suggest that baseline tumor immune activation status may represent the core molecular determinant of ICI efficacy. Correspondingly, tumor tissue PD-L1 expression levels have been validated across multiple studies to predict ICI treatment response—high PD-L1 expression correlates with better response to anti-PD-1 antibodies such as nivolumab and pembrolizumab, establishing it as a histopathological basis for screening ICI candidates (66). Notably, CTNNB1 (β-catenin) mutations can influence immunotherapy sensitivity through Wnt pathway activation, while TERT promoter and TP53 mutations are closely associated with adverse prognosis and treatment resistance; these genomic alterations can be detected via NGS analysis of preoperative biopsies (67, 68). However, all aforementioned biomarkers rely on high-throughput sequencing technologies, which are costly and complex, and all studies encompassed fewer than 50 samples lacking validation in independent cohorts, remaining distant from clinical application.

For traditional locoregional and systemic therapies such as HAIC, evidence supporting the predictive value of serum biomarkers is more mature. Baseline AFP levels have been consistently validated across multiple studies as an important predictor of HAIC (FOLFOX regimen) efficacy, with AFP ≥400 ng/mL indicating poorer prognosis and serving as an independent predictor of PFS (53, 54). Building on this foundation, a composite model incorporating AFP and C-reactive protein (CRP) further enhanced predictive performance (53), while plasma DPP4/CD26 levels (cutoff value 164.6 ng/mL) were validated as independent protective factors for OS and PFS (52). In the broader systemic therapy field, dynamic changes in AFP similarly carry important value for efficacy monitoring: AFP decrease >50% following TACE or radiotherapy significantly correlates with imaging response, and AFP decrease >20% following systemic chemotherapy likewise associates with superior survival outcomes (69). However, the value of AFP in predicting sorafenib response remains controversial—although Nakazawa et al. reported that AFP elevation >20% within four weeks of sorafenib associates with shorter survival, Llovet et al. found that AFP changes from baseline to week 12 do not predict survival, with imaging currently remaining the gold standard for assessing targeted therapy response (69). AFP-L3, the fucosylated isoform of AFP, provides more precise prognostic stratification: patients with post-operative AFP-L3 <15% achieved 5-year survival of 91.7%, compared to only 23.8% for those ≥15% (70). At the targeted therapy mechanism level, amplification of fibroblast growth factors 3 and 4 (FGF3/FGF4) was shown to predict sorafenib response—30% of partial or complete responders harbor this amplification compared to zero detection in non-responders (71). High c-Met expression was similarly validated to predict therapeutic benefit from Met inhibitor tivantinib, with significantly superior median time to progression in high expressers versus low expressers (2.7 months vs 1.4 months, HR 0.43, p=0.03) (72). Among circulating biomarkers, baseline angiopoietin-2 (Ang-2) and VEGF levels independently mark disease progression velocity but do not directly predict sorafenib response (73).

Recently, liquid biopsy centered on ctDNA and CTCs has provided emerging non-invasive dimensions for efficacy prediction. EpCAM-positive CTCs represent the most well-established post-operative recurrence predictor: ≥2 EpCAM+ CTCs in 7.5 mL pre-operative blood represents the strongest independent predictor of hepatic resection recurrence (74). The proportion of mesenchymal-type CTCs closely correlates with early recurrence and distant metastasis (75). Regarding ctDNA, RASSF1A gene promoter hypermethylation is detectable in 93% of HCC patients, with high concentration associating with poor disease-free survival (76). Yu and Ma’s review further notes that dynamic ctDNA monitoring demonstrates promise in detecting minimal residual disease and predicting recurrence (77, 78). Overall, these serum and circulating molecular biomarkers offer convenient detection, yet their predictive mechanisms remain unclear, the generalizability of cutoff values awaits prospective validation, and the extreme rarity of CTCs and low concentration of ctDNA present challenges for detection technologies.

### 6.2 Cellular-level biomarkers

Cellular-level research has revealed two mutually corroborating research directions: CD8^+^ T-cell infiltration within tumors represents the most consistently identified positive predictive factor across studies and treatment regimens, while enrichment of immunosuppressive myeloid cells is closely associated with treatment resistance. In recent years, the research perspective has shifted from absolute enumeration of individual cell subsets toward spatial relationships between cells, representing an important methodological advance in this field.

Regarding T cells, mass cytometry confirmed that patients achieving ≥50% pathological necrosis with cemiplimab exhibited significantly higher intratumoral CD8^+^ T-cell infiltration compared to non-responders (32). Those attaining MPR with nivolumab ± Ipilimumab showed significantly increased frequency of activated CD8^+^CD45RO^+^CD57^+^Eomes^+^CD38^low effector T cells from baseline (34). At the peripheral blood level, T-cell clonal expansion following tislelizumab combined with SBRT treatment positively correlated with treatment efficacy (63). Tumor-infiltrating lymphocytes (TILs) as a key biomarker reflecting host antitumor immune response, high levels associate with better prognosis and immunotherapy response; TIL analysis facilitates prediction of treatment outcomes and guides combination immunotherapy strategies (79, 80). These findings collectively support the central role of T cells in treatment response prediction from both tumor microenvironment and peripheral circulation dimensions.

In contrast to the positive predictive role of T cells, VISTA^+^ myeloid cell populations (CD68^+^CD11b^+^CD11c^+^VISTA^+^) were significantly enriched in non-MPR patients, suggesting that VISTA-mediated immune suppression represents an important mechanism of ICI resistance (34). More methodologically instructive, Ho et al., employing imaging mass cytometry combined with random forest algorithms, discovered that the determinant of cabozantinib plus nivolumab efficacy is not absolute cell number but rather spatial proximity relationships between cells: proximity of T/B cells to proliferative PD-L1^+^ macrophages coupled with distance from immunosuppressive Arginase-1^+^ macrophages predicted response. The opposite spatial arrangement predicted resistance (42). This finding suggests that the functional state of the tumor microenvironment is determined by both cellular composition and spatial architecture. However, high-dimensional single-cell technologies are currently applicable only in research settings, with inconsistent definitions of cell subsets across different studies.

### 6.3 Histopathological-level biomarkers

Core findings at the histopathological level can be summarized as follows: the presence of tertiary lymphoid structures (TLS) and baseline immune infiltration density represent consistent positive predictive factors for ICI efficacy, with both likely reflecting the “hot” and “cold” states of the tumor immune microenvironment.

TLS, as lymphocyte aggregates harboring germinal centers, have been independently validated across multiple studies to correlate with treatment efficacy. Responder tumors treated with cabozantinib plus nivolumab contained significantly more TLS compared to non-responders (P = 0.006) (42), and TLS formation induced by nivolumab plus ipilimumab similarly associated with enhanced antitumor immunity (37). However, TLS assessment currently lacks unified quantitative standards, affecting result reproducibility.

Baseline immune infiltration density not only predicts efficacy but may also guide treatment regimen selection. Kaseb et al.’s study demonstrated that high baseline density of T cells, B cells, and cytotoxicity markers represents a prerequisite for nivolumab monotherapy to achieve MPR, while patients with low baseline immune infiltration require combination with ipilimumab to induce MPR (34). Marron et al. further confirmed through multiplex immunohistochemistry that patients with higher baseline immune infiltration were more likely to achieve ≥50% tumor necrosis (32). Beyond immune microenvironment architecture, traditional histopathological biomarkers provide important background information for pathological assessment following neoadjuvant therapy. Heat shock protein 70 (HSP70) is significantly upregulated in early HCC tissue, with overexpression correlating to portal vein invasion, cellular proliferation, and larger tumor size; glutamine synthetase (GS) exhibits diffuse expression in hepatocellular tumors and correlates with invasive tumor characteristics; combined positivity of both aids in distinguishing well-differentiated HCC (81, 82). Overall, histopathological biomarkers depend on biopsy specimens and carry sampling bias risk, and current studies have not established externally validated immune infiltration scoring systems.

### 6.4 Imaging-level biomarkers

Imaging biomarkers possess unique advantages due to their non-invasive nature and reproducibility, yet direct evidence in this field is currently more limited. Existing literature simultaneously indicates that traditional RECIST 1.1 criteria and its modified mRECIST version have systematic limitations in targeted therapy and immunotherapy contexts—treatment-induced tumor necrosis does not necessarily accompany volume reduction, leading to systematic underestimation of efficacy. AFP is similarly not considered a standard measure of sorafenib response, with imaging being the currently recognized standard of evaluation (73).

In Marron et al.’s study, quantitative assessment of tumor necrotic regions (non-enhancing tissue) on contrast-enhanced MRI significantly correlated with pathological necrosis degree (r=0.62, P = 0.0049), and was superior to conventional RECIST 1.1 criteria (32). This finding suggests that MRI functional imaging may address deficiencies in morphological assessment standards. However, the aforementioned evidence derives from only a single-arm, small-sample study, standardized protocols for MRI necrosis assessment have not been established, and the potential of radiomics and artificial intelligence-assisted analysis in this field awaits systematic exploration.

### 6.5 AI and machine learning-enhanced biomarker development

Beyond conventional radiomics, artificial intelligence (AI) and machine learning (ML) methods are increasingly being applied to enhance biomarker discovery and predictive modeling in HCC. These approaches leverage high-dimensional imaging data to construct predictive models that outperform traditional single-marker strategies (83). In the context of treatment response prediction, CT-based machine learning radiomics models have demonstrated high accuracy for predicting response to lenvatinib plus PD-1 inhibitors combined with interventional therapy, achieving an AUC of 0.893 in independent validation (84). Multiphase deep learning models integrating plain scan, arterial, and portal venous phase CT images further improved predictive performance for lenvatinib and immune checkpoint inhibitor combinations, with interpretable heatmaps identifying necrosis, vasculature, and tumor heterogeneity as critical response-determining features.99 In the perioperative setting, radiomic analysis of preoperative contrast-enhanced CT has shown robust performance in predicting microvascular invasion and postoperative recurrence, providing a non-invasive tool for preoperative risk stratification in resectable HCC (85). Machine learning-based radiomic models have been shown to outperform conventional clinical biomarkers in predicting immunotherapy outcomes, highlighting the added value of AI-driven imaging biomarkers beyond established laboratory and clinical parameters (86). Furthermore, multimodal fusion systems that integrate CT imaging, clinical variables, and laboratory parameters have demonstrated superior predictive accuracy for immune checkpoint inhibitor survival benefits compared to single-modality models, underscoring the potential of data integration in personalized treatment planning (87). Despite these promising results, most AI models in HCC have been developed and validated in single-center, retrospective cohorts with limited external validation, raising concerns about generalizability across diverse populations and imaging protocols.104 (88) Future efforts should prioritize multi-center prospective validation, standardized imaging protocols, and the development of clinically deployable models that can be seamlessly integrated into radiology workflows.

## 7 Discussion

HCC is a highly lethal malignancy globally, often diagnosed at intermediate or advanced stages, with low survival rates in most countries, including high-income nations where no significant improvement in survival has been observed (89). Currently, the main curative treatments for HCC are surgical resection and liver transplantation (90). However, even after curative treatment for early-stage HCC, the 5-year recurrence rate is as high as 70% (91). To address these challenges, various therapeutic strategies have been explored in the past, with advancements in adjuvant therapy providing key support for perioperative management. Studies such as IMbrave050 have achieved significant clinical breakthroughs, but the median overall survival remains less than 2 years (92), resistance is widespread, and treatment options for special populations such as those with PVTT Vp4 are still limited (93), suggesting that HCC treatment has entered a relatively stagnant phase. Therefore, promoting the application of neoadjuvant therapy has become an important research direction.

Neoadjuvant therapy represents the strategic preoperative application of systemic or locoregional treatment in patients with resectable HCC, where the primary goal is to eradicate micrometastatic disease, improve R0 resection rates, and prolong long-term survival. The efficacy of neoadjuvant approaches in this setting is shaped by a complex interplay of factors, including underlying liver function reserve, portal hypertension status, performance status, tumor burden and vascular involvement, and the molecular and immune characteristics of the tumor microenvironment (94). Importantly, the influence of these multidimensional variables on treatment outcomes is not unique to neoadjuvant therapy. Rather, it reflects the fundamental challenge of delivering systemic and locoregional treatments to a patient population characterized by both oncologic heterogeneity and compromised hepatic functional reserve. The clinical value of neoadjuvant therapy in resectable HCC has been demonstrated across multiple treatment modalities. For instance, in patients with resectable tumors, TACE combined with sintilimab as neoadjuvant therapy achieved an ORR of 62% and a pCR rate of 14% (95). Nivolumab combined with ipilimumab demonstrated a MPR rate of 27%, with no recurrences observed in MPR patients during a 24.6-month follow-up (34). The FOLFOX-HAIC regimen brought a 3-year survival rate of 77.1% (54). In patients with multifocal tumors or PVTT Vp1-2, the combination of lenvatinib, PD-1 inhibitors, and TACE achieved a pCR rate of 25% (49). These data collectively support the potential of neoadjuvant therapy to improve outcomes in resectable HCC.

**Figure 3:**
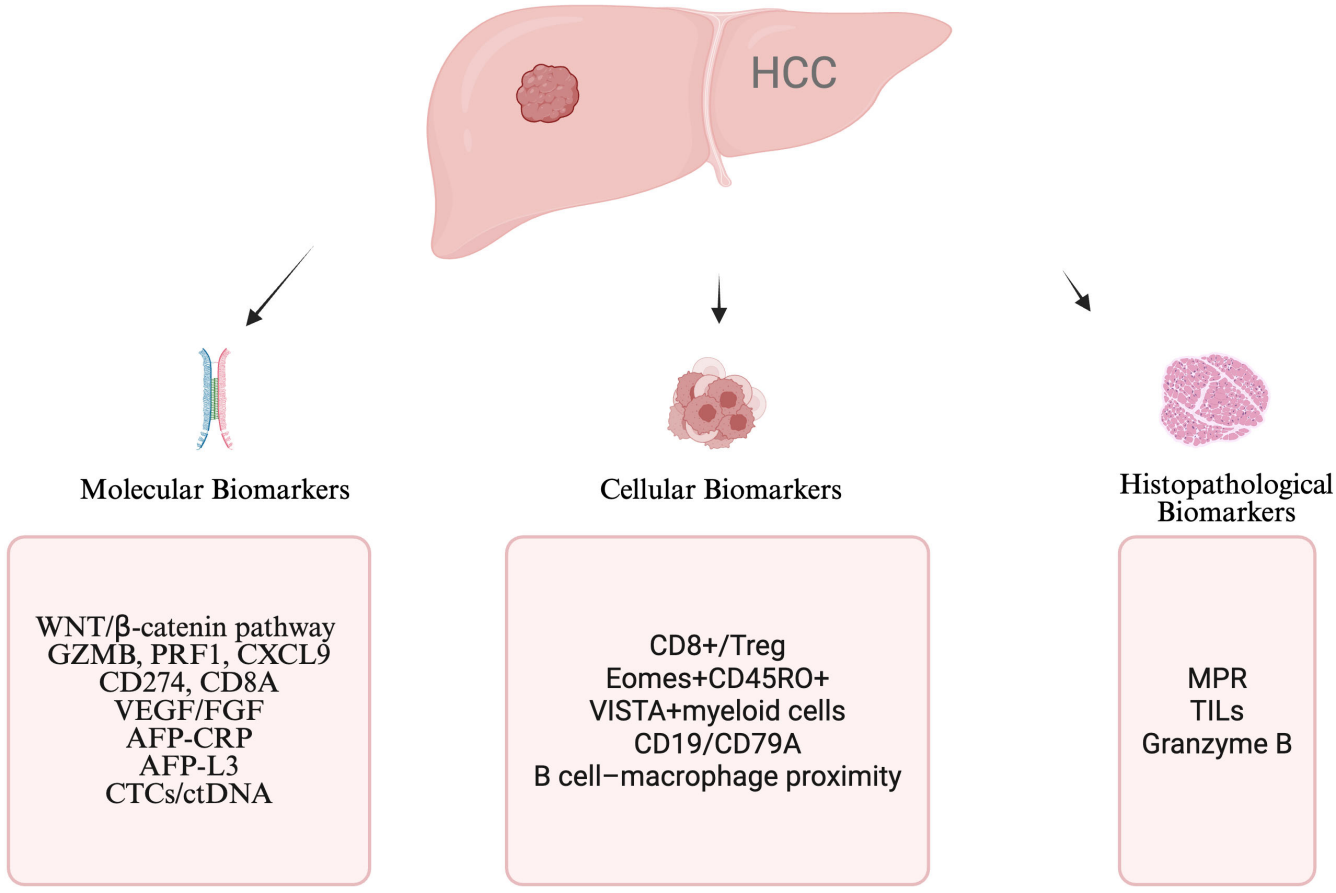
Classification of Predictive Biomarkers for Neoadjuvant Therapy in HCC. This figure summarizes key predictive biomarkers for neoadjuvant therapy in hepatocellular carcinoma across molecular, cellular, and histopathological dimensions. Molecular biomarkers include negative predictors such as WNT/β-catenin pathway activation for ICIs, positive indicators of immune activation including T-effector gene signatures (GZMB, PRF1, CXCL9) and CD274 (PD-L1) expression, angiogenic factors (VEGF/FGF) for targeted therapy response, serum markers (AFP, AFP-L3, AFP-CRP composite model) for locoregional and systemic therapy monitoring, and circulating biomarkers (CTCs, ctDNA) for post-treatment recurrence prediction. Cellular biomarkers feature immune profiles such as CD8+ T-cell/Treg ratios, activated effector T-cell phenotypes (Eomes+CD45RO+), VISTA+ myeloid cell enrichment associated with resistance, CD19/CD79A B-cell maturation markers, and the spatial proximity of B cells to specific macrophage subsets as determinants of treatment response. Histopathological assessment highlights major pathological response (MPR), tumor-infiltrating lymphocytes (TILs), and granzyme B expression as indicators of treatment efficacy and immune activation. The evolution from conventional serum markers to multi-dimensional molecular, cellular, and spatial biomarkers reflects the ongoing pursuit of reliable tools to guide personalized neoadjuvant treatment, though challenges in standardization, prospective clinical validation, and integration into routine practice persist.

Another major advantage of neoadjuvant therapy is its role as an “*in vivo* drug sensitivity testing platform” (96), providing a basis for the selection of postoperative adjuvant therapy strategies. Biomarker analysis holds certain value in assessing the response to neoadjuvant therapy. Previous studies have made some progress in this area. Tumor immune microenvironment features, such as the formation of tertiary lymphoid structures, combined with peripheral blood markers like circulating tumor DNA (ctDNA) dynamics, can effectively predict the pathological response and long-term survival benefits of immunotherapy combinations, providing important evidence for clinical treatment decisions (26). It is important to note that the biomarkers discussed in this review—including T-effector gene signatures (GZMB, PRF1, CXCL9), PD-L1 expression, Wnt/β-catenin pathway alterations, and circulating markers such as AFP and ctDNA—were originally identified and validated predominantly in the context of advanced-stage HCC treated with systemic immunotherapy or targeted therapy (97). Their predictive utility extends across the disease stage continuum from advanced to resectable HCC, reflecting shared biological determinants of treatment response rather than stage-specific mechanisms. This continuity underscores the translational relevance of biomarker research conducted in advanced HCC to the neoadjuvant setting, though optimal cutoff values and integration strategies may require stage-specific refinement. Notably, a paradigm shift is emerging in biomarker utilization, moving beyond static thresholds like AFP levels toward dynamic models that capture real-time tumor-immune interactions (98). For instance, CXCL13^+^CD4^+^ T cell infiltration and CD8^+^/Treg ratios reflect adaptive immune activation, while spatial features such as B cell-macrophage proximity predict response to combination therapies (**Figure 3**) (99). Nonetheless, in clinical practice, a comprehensive evaluation of these predictive factors is still necessary, primarily due to a lack of robust clinical evidence.

Therefore, a comprehensive evaluation of these predictive factors remains essential in clinical practice. Most existing studies are small-scale phase 1/2 trials with limited follow-up, and insufficient high-level evidence demonstrating durable survival benefits to justify routine adoption. Safety concerns also persist, particularly with immunotherapy: immune checkpoint inhibitors may trigger severe immune-related adverse events, posing significant risks for HCC patients with compromised liver function or portal hypertension. Uncertainties further exist in efficacy assessment, as short-term metrics like pathological response or radiological tumor shrinkage have not been rigorously validated as surrogates for long-term survival in HCC. The lack of harmonized pathological response criteria—exemplified by variable MPR thresholds ranging from ≥50% to ≥90% tumor necrosis across trials—complicates cross-study comparisons and limits the regulatory utility of pathological endpoints. Inconsistent definitions of pCR and MPR, divergent specimen sampling protocols, and the absence of immune-adapted criteria that account for treatment-induced changes in the tumor microenvironment further underscore the need for international consensus. In addition, the absence of reliable, prospectively validated biomarkers complicates the identification of patients who truly benefit from neoadjuvant therapy. Thus, the clinical application of neoadjuvant therapy in resectable HCC warrants further investigation through well-designed prospective trials.

Looking ahead, several high-priority research directions warrant focused investment. First, the development of integrative biomarker frameworks that combine spatial transcriptomics, imaging mass cytometry, and liquid biopsy represents a critical frontier. Rather than relying on single-marker thresholds, future studies should pursue composite scoring systems that weight baseline immune activation signatures (IFN-γ signaling, T-effector gene expression), spatial microenvironment architecture (T/B cell proximity to antigen-presenting macrophages), and dynamic ctDNA kinetics to generate individualized response probability estimates. The clinical translation of such multidimensional models will require prospective validation in biomarker-enriched trial designs, where treatment allocation is informed by real-time molecular profiling rather than conventional staging alone. Second, the standardization of pathological response criteria in HCC demands urgent attention. While MPR and pCR have emerged as candidate surrogate endpoints, the threshold heterogeneity across trials—ranging from >50% to ≥90% tumor necrosis—limits comparability and regulatory acceptance. Future efforts should establish harmonized definitions through international pathology consensus conferences, analogous to the IASLC initiatives in lung cancer. Beyond conventional necrosis-based metrics, immune-adapted response criteria incorporating treatment-induced lymphoid aggregates, immune cell infiltration patterns, and tertiary lymphoid structure density may provide biologically richer endpoints that better capture the mechanistic basis of immunotherapy efficacy in HCC. Third, the optimization of neoadjuvant–adjuvant sequencing strategies represents a compeling but underexplored domain. Emerging evidence from melanoma and lung cancer suggests that perioperative immunotherapy may outperform adjuvant-only approaches by priming *de novo* antitumor immunity while the primary tumor remains *in situ*. However, the optimal duration of neoadjuvant treatment, the timing of surgical intervention relative to peak immune activation, and the need for postoperative therapy intensification or de-escalation based on pathological response status remain undefined in HCC. Trials incorporating window-of-opportunity designs—with detailed immune profiling at baseline, mid-treatment, and post-resection timepoints—could elucidate the kinetics of immune priming and inform adaptive treatment algorithms. Fourth, radiomics and artificial intelligence offer transformative potential for non-invasive response prediction in the neoadjuvant setting. Machine learning models integrating pre-treatment MRI texture features, quantitative perfusion parameters, and clinical variables have demonstrated promising accuracy for predicting pathological response in preliminary studies. The convergence of radiomics with genomic and transcriptomic data—termed “radiogenomics”—may enable non-invasive mapping of intratumoral molecular heterogeneity, guiding both patient selection and real-time treatment adaptation without repeated biopsy. Finally, the integration of ctDNA-based molecular residual disease monitoring into perioperative management frameworks warrants prospective evaluation. Serial ctDNA assessment during neoadjuvant therapy could serve as an early efficacy signal, enabling treatment escalation for molecular non-responders and potentially sparing unnecessary toxicity for patients with rapid ctDNA clearance. Postoperatively, ctDNA dynamics may identify patients at high risk of recurrence who would benefit from intensified adjuvant strategies, while ctDNA-negative patients might be candidates for de-escalated surveillance. Realizing this vision will require standardized ctDNA assay platforms, validated threshold definitions, and integration with imaging-based surveillance protocols in prospective clinical trials.

## 8 Conclusion

Neoadjuvant therapy is emerging as a transformative strategy to improve surgical outcomes and reduce recurrence in resectable HCC, particularly through the integration of immunotherapy-based systemic regimens with locoregional modalities. The highest level of evidence to date comes from the phase 2/3 CARES-009 trial, which demonstrated significant event-free survival benefit with perioperative camrelizumab plus rivoceranib, while phase 3 data for neoadjuvant HAIC have established locoregional therapy as a benchmark in resectable BCLC A/B disease beyond Milan criteria. Recent advances in combination regimens have demonstrated encouraging pathological response rates, with MPR achievement consistently associated with improved recurrence-free survival across multiple trials. Nevertheless, substantial challenges persist: the heterogeneity of underlying liver disease and portal hypertension introduces treatment-effect variability that is not unique to neoadjuvant approaches but demands careful patient selection; pathological response criteria remain unharmonized across trials; and validated biomarkers for guiding treatment decisions are lacking. Future progress will depend on the development of integrative biomarker frameworks combining spatial multi-omics, ctDNA dynamics, and radiogenomic signatures; the establishment of standardized immune-adapted pathological endpoints; and the optimization of neoadjuvant–adjuvant sequencing through window-of-opportunity trials with detailed immune profiling. Through these concerted efforts, neoadjuvant therapy holds the potential to redefine curative-intent management for resectable HCC in the era of precision perioperative oncology.

## Glossary

3DCRT: 3D Conformal Radiotherapy
AASLD: American Association for the Study of Liver Diseases
AFP: Alpha-Fetoprotein
ALD: Alcohol-Associated Liver Disease
ALT/AST: Alanine Aminotransferase/Aspartate Aminotransferase
AUC: Area Under the Curve
BCLC: Barcelona Clinic Liver Cancer
CD8+/Treg: CD8+ T cell to Regulatory T cell ratio
CNLC: China Liver Cancer
CTLA-4: Cytotoxic T-lymphocyte-associated protein-4
DC: Dendritic Cell
DCR: Disease Control Rate
DEB-TACE: Drug-Eluting Bead Transarterial Chemoembolization
DFS: Disease-Free Survival
E2F/MYC: E2 Transcription Factor/Myelocytomatosis Oncogene
EFS: Event-Free Survival
ESMO: European Society for Medical Oncology
HAIC: Hepatic Arterial Infusion Chemotherapy
HBV: Hepatitis B Virus
HCC: Hepatocellular Carcinoma
HCV: Hepatitis C Virus
HIF-1α: Hypoxia-Inducible Factor-1α
HR: Hazard Ratio
ICI: Immune Checkpoint Inhibitor
ICIs: Immune Checkpoint Inhibitors
IMRT: Intensity-Modulated Radiotherapy
IPCR: Image-Pathology Concordance Rate
MASLD: Metabolic Dysfunction-Associated Steatotic Liver Disease
MDT: Multidisciplinary Team
MPR: Major Pathological Response
MVI: Microvascular Invasion
MWA: Microwave Ablation Therapy
NASH: Non-Alcoholic SteatoHepatitis
NCCN: National Comprehensive Cancer Network
ORR: Objective Response Rate
OS: Overall Survival
PD-1: Programmed Death-1
PD-L1: Programmed Death-Ligand 1
PFS: Progression-Free Survival
PR: Partial Response
PVTT: Portal Vein Tumor Thrombosis
R0: R0 resection (complete resection with no microscopic residual tumor)
RECIST: Response Evaluation Criteria in Solid Tumors
RFA: Radiofrequency Ablation
RFS: Recurrence-Free Survival
RR: Response Rate
RT: Radiotherapy
SBRT: Stereotactic Body Radiotherapy
SD: Stable Disease
SIRT: Selective Internal Radiation Therapy
TACE: Transarterial Chemoembolization
TIL: Tumor-Infiltrating Lymphocyte
TILs: Tumor-Infiltrating Lymphocytes
TKI: Tyrosine Kinase Inhibitor
TKIs: Tyrosine Kinase Inhibitors
TLS: Tertiary Lymphoid Structures
TME: Tumor Microenvironment
VEGF: Vascular Endothelial Growth Factor
VEGFR2/PDGFR: VEGF Receptor 2/Platelet-Derived Growth Factor Receptor
VISTA: V-domain Ig Suppressor of T cell Activation
cTACE: Conventional Transarterial Chemoembolization
ctDNA: Circulating Tumor DNA
mRECIST: modified Response Evaluation Criteria in Solid Tumors
pCR: Pathological Complete Response
